# Ablation of Endothelial TRPV4 Channels Alters the Dynamic Ca^2+^ Signaling Profile in Mouse Carotid Arteries

**DOI:** 10.3390/ijms21062179

**Published:** 2020-03-22

**Authors:** Stuart J. McFarland, David S. Weber, Chung-sik Choi, Mike T. Lin, Mark S. Taylor

**Affiliations:** Department of Physiology and Cell Biology, University of South Alabama College of Medicine, Mobile, AL 36688, USA; sjm1721@jagmail.southalabama.edu (S.J.M.); dweber@southalabama.edu (D.S.W.); cchoi@southalabama.edu (C.-s.C.); mlin@southalabama.edu (M.T.L.)

**Keywords:** transient receptor potential channels, TRPV4, calcium, endothelium

## Abstract

Transient receptor potential vanilloid 4 channels (TRPV4) are pivotal regulators of vascular homeostasis. Altered TRPV4 signaling has recently been implicated in various cardiovascular diseases, including hypertension and atherosclerosis. These versatile nonselective cation channels increase endothelial Ca^2+^ influx in response to various stimuli including shear stress and G protein-coupled receptor (GPCR) activation. Recent findings suggest TRPV4 channels produce localized Ca^2+^ transients at the endothelial cell plasma membrane that may allow targeted effector recruitment and promote large-scale Ca^2+^ events via release from internal stores (endoplasmic reticulum). However, the specific impact of TRPV4 channels on Ca^2+^ signaling in the intact arterial intima remains unknown. In the current study, we employ an endothelium-specific TRPV4 knockout mouse model (ecTRPV4^−/−^) to identify and characterize TRPV4-dependent endothelial Ca^2+^ dynamics. We find that carotid arteries from both ecTRPV4^−/−^ and WT mice exhibit a range of basal and acetylcholine (ACh)-induced Ca^2+^ dynamics, similar in net frequency. Analysis of discrete Ca^2+^ event parameters (amplitude, duration, and spread) and event composite values reveals that while ecTRPV4^−/−^ artery endothelium predominantly produces large Ca^2+^ events comparable to and in excess of those produced by WT endothelium, they are deficient in a particular population of small events, under both basal and ACh-stimulated conditions. These findings support the concept that TRPV4 channels are responsible for generating a distinct population of focal Ca^2+^ transients in the intact arterial endothelium, likely underlying their essential role in vascular homeostasis.

## 1. Introduction

Dynamic Ca^2+^ signals direct many aspects of endothelial function and hence vascular homeostasis. They control the degree and specificity of many responses from vasodilation to permeability and inflammation. In endothelial cells, discrete Ca^2+^ signals emit from internal Ca^2+^ stores (i.e., release from the endoplasmic reticulum through inositol 1,4,5-trisphosphate receptors; IP_3_Rs) and along the plasma membrane (i.e., influx through transient receptor potential (TRP) channels) in the form of transients and waves [1,2]. Recent efforts to dissect endothelial Ca^2+^ dynamics through automated detection and analysis approaches have revealed that spatial and temporal tuning of basally occurring Ca^2+^ dynamics drives graded stimulus responses (e.g., vasodilation) [3]. As key contributors to endothelial Ca^2+^ influx, type 4 vanilloid transient receptor potential (TRPV4) channels have emerged as crucial regulators of endothelial function and as pivotal players in the development of cardiovascular disease [4,5,6,7,8]. These nonselective cation channels are versatile environmental detectors, integrating various chemical and physical stimuli, including receptor agonist second messengers (e.g., arachidonic acid metabolites), temperature, and shear stress to affect a range of Ca^2+^-dependent pathways [2,9,10,11,12,13,14]. TRPV4 channels expressed in smooth muscle and endothelial cells control vascular tone and permeability. In the endothelium, these channels act through Ca^2+^-activated K^+^ channels (K_Ca_) to elicit membrane potential hyperpolarization that can spread to smooth muscle and promote vasorelaxation [2,6]. Endothelial hyperpolarization may also enhance TRPV4 Ca^2+^ influx, allowing widespread expansion of Ca^2+^ dynamics (i.e., through Ca^2+^-induced Ca^2+^ release from IP_3_Rs) [15,16]. 

Dysregulation of TRPV4 signaling contributes to endothelial dysfunction and progressive disease. Diabetes and atherosclerosis are associated with loss of functional endothelial TRPV4 channels [17], and multiple animal models of hypertension have recently been linked to loss of interaction between TRPV4 and K_Ca_2.3 channel subunits [6,18]. On the other hand, increased TRPV4 activity in the lungs under conditions of positive ventilation pressure leads to increased endothelial permeability and pulmonary edema [19]. These findings attest to the clinical prominence of endothelial TRPV4 channels and emphasize the need to better understand their influence on cell signaling. 

Recent studies suggest influx through single or small clusters of TRPV4 channels give rise to highly localized Ca^2+^ transients (or sparklets) along the endothelial cell plasma membrane [20]. Occurrence of these events is potentiated by direct pharmacologic activation of TRPV4 channels or GPCRs. Notably, in mouse mesenteric arteries, stimulation with the TRPV4 agonist GSK-1016790A or muscarinic receptors with ACh elicited TRPV4 sparklets when internal Ca^2+^ stores were depletion [2]. However, under physiologic conditions in which internal stores remained intact, both stimuli produced a wide range of endothelium Ca^2+^ dynamics and waves. This suggests that in intact vascular endothelium, fundamental TRPV4 Ca^2+^ events can be actively recruited, and they may act as triggers for much larger Ca^2+^ signals. In the current study, we employ a genetically altered mouse deficient in endothelial TRPV4 to determine the specific impact of TRPV4 channels on basal and stimulated Ca^2+^ signaling profiles in the arterial endothelium. 

## 2. Results 

### 2.1. Generation of Endothelium-Specific Knockout Mice (ecTRPV4^−/−^) and Assessment of Arterial Expression

**Figure 1:**
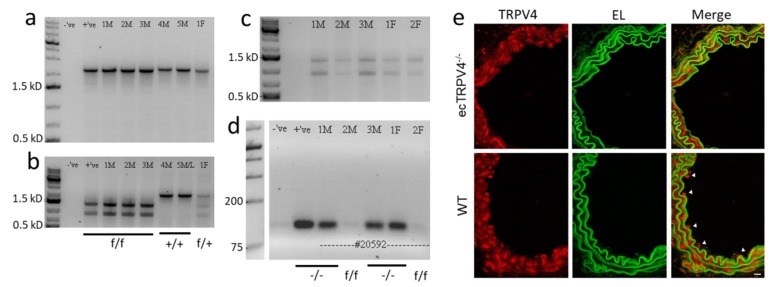
Endothelium-specific TRPV4 knockout mouse genotyping and arterial expression. (**a**) DNA genotyping and PCR products of F2 pups. (**b**) TRPV4-floxed allele contains a PacI digestion site, which is absent in the wild-type (WT) allele. Subsequent PacI digestion of the PCR products reveals mouse genotypes—WT (+/+), heterozygous (f/+) and homozygous TRPV4-floxed (f/f) mice. (**c**) PCR products obtained from F3 pups after PacI digestion. All mice were TRPV4f/f. (**d**) The same mice were further genotyped for the endothelium-specific CRE PCR product. Mice that express TRPV4f/f and CRE were selected as breeders to create TRPV4 knockout (−/−) mice. (**e**) Representative images of TRPV4 immunofluorescence in ecTRPV4^−/−^ and WT mouse carotid arteries. TRPV4-positve endothelial staining was observed in WT (arrowheads) but not ecTRPV4^−/−^ arteries. EL, elastic lamina; Scale bar, 10 µm.

Figure 1a–d shows the progressive strategy for creating ecTRPV4^−/−^ breeders by generating and crossing floxed TRPV4 mice with endothelial-specific CRE. Assessment of TRPV4 expression in carotid arteries isolated from ecTRPV4^−/−^ mice and control wild-type (WT) mice (Figure 1e) reveals TRPV4-positive fluorescence in endothelial cells along the vascular intima of WT arteries (inside the internal elastic lamina), whereas the signal was essentially absent along the ecTRPV4^−/−^ artery intima. Positive staining was evident in the medial and adventitial layers of both ecTRPV4^−/−^ and WT mice.

### 2.2. Basal and Acetylcholine-Induced Ca^2+^ Dynamics along the Intima of ecTRPV4^−/−^ Carotid Arteries

**Figure 2:**
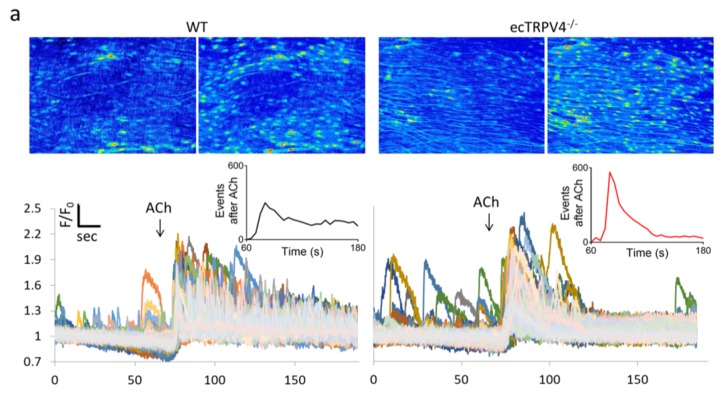

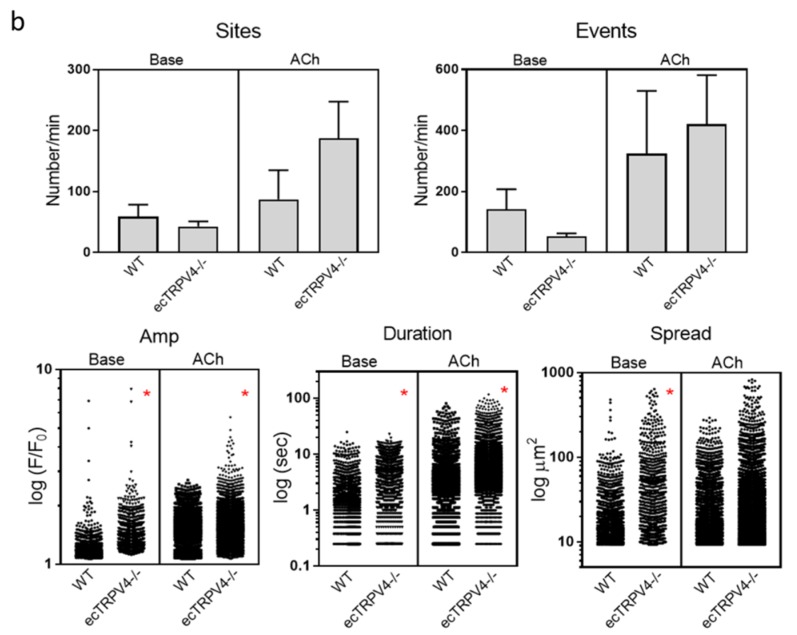
Endothelial Ca^2+^ dynamics in ecTRPV4^−/−^ and WT mouse carotid arteries. (**a**) Opened carotid arteries isolated from wild-type control mice (WT, left) and endothelium-specific TRPV4 knockout (ecTRPV4^−/−^, right) mice were loaded with fluorescent Ca^2+^ indicator Fluo-4 AM and assessed via spinning disk confocal before and after addition of ACh (10^−8^ M). Maximum projection images of time-lapse recordings (top) and corresponding continuous recordings (bottom) are shown (20× magnification). Inserts show the time course of events after ACh exposure (for all experiments, *n* = 8). (**b**) Bar graphs show the number of Ca^2+^ signaling sites and events occurring per minute in WT and ecTRPV4^−/−^ artery endothelia before and after ACh exposure. Individual event parameters (amplitude, duration and spatial spread) are displayed in the corresponding scatterplots. *n* = 8; * indicates *p* < 0.05 for ecTRPV4^−/−^ compared to respective WT.

**Figure 3:**
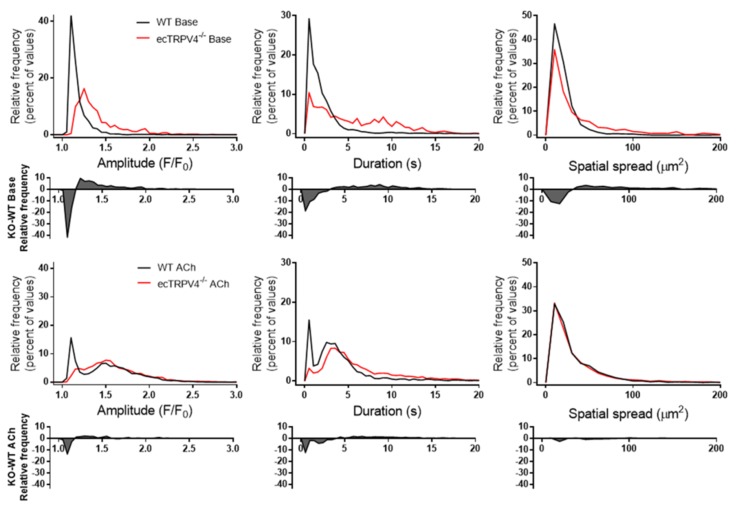
Separation of ecTRPV4^−/−^ and WT Ca^2+^ event parameter distributions. Histograms show relative frequency distributions of amplitude, duration and spatial spread values under basal (top) and ACh-stimulated (10^−8^ M) conditions (bottom). Shaded plots show difference between ecTRPV4^−/−^ and WT histograms for each parameter (KO-WT).

In order to determine the specific impact of TRPV4 ablation on the dynamic endothelial Ca^2+^ signaling profile, we assessed the intima of opened carotid artery segments loaded with the Ca^2+^ indicator Fluo-4. Figure 2a shows confocal images and time-lapse recordings from ecTRPV4^−/−^ and WT mouse carotid artery endothelium before and after exposure to acetylcholine. The ecTRPV4^−/−^ artery endothelium exhibited a similar number of Ca^2+^ signal origination sites and events as WT artery endothelium, both basally and after 10 nM ACh stimulation (Figure 2b). While the highest density of events occurred within 20 s of ACh exposure in both WT and ecTRPV4^−/−^ vessels, events continued to fire consistently over 2 min in the WT endothelium, whereas activity was notably muted after 60 s in the ecTRPV4^−/−^ endothelium. Overall, ecTRPV4^−/−^ endothelium events displayed larger event amplitude, duration and spatial spread than WT events (median values 1.14 vs. 1.32 F/F_0_, 1.25 vs. 4.25 s, and 47 vs. 66 µm^2^, respectively). Following ACh exposure, event amplitudes and durations remained higher in ecTRPV4^−/−^ endothelium (median values 1.48 vs. 1.54 F/F_0_, 3.12 vs. 4.88 s, respectively), whereas spatial spread was not significantly different than WT (62 vs. 64 µm^2^). Histogram analysis revealed a rightward displacement of the ecTRPV4^−/−^ event parameter distributions relative to WT (Figure 3). Difference plots revealed a reduced number of events with amplitude < 1.2 F/F_0_, duration < 3.4 s, and spread < 36 µm^2^ in ecTRPV4^−/−^ vs. WT endothelium. Conversely, there was a modest increase in the number of events with parameters above these values in ecTRPV4^−/−^ vs. WT. Notably, after ACh stimulation, ecTRPV4^−/−^ and WT histograms became increasingly convergent, but the paucity of small amplitude and small duration events persisted in ecTRPV4^−/−^ endothelium. 

### 2.3. Composite Assessment of Ca^2+^ Signal Profiles in ecTRPV4^−/−^ Arteries

**Figure 4:**
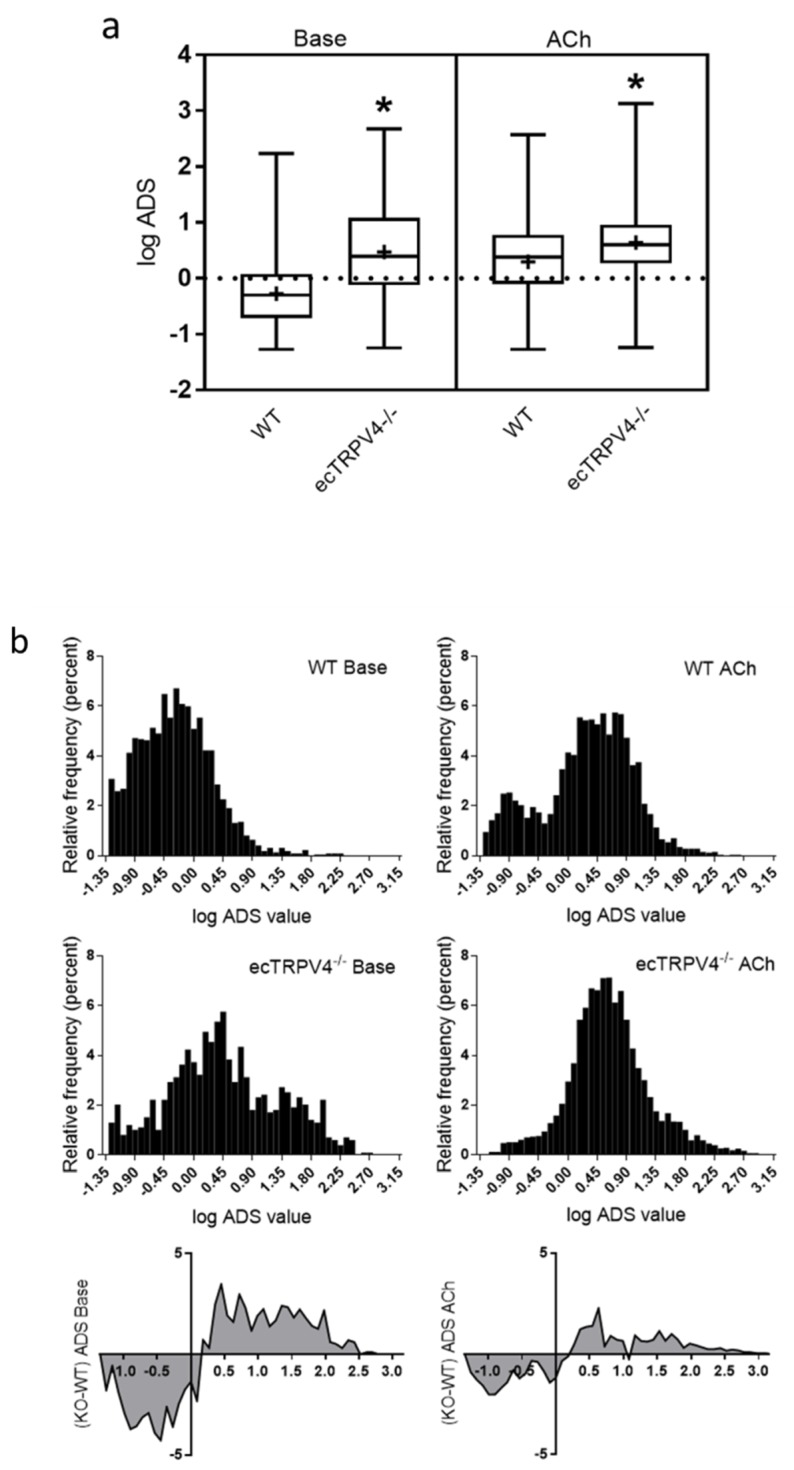
Holo-Ca^2+^ event profiling using ADS composite distributions. (**a**) Box-and-whisker plots of log ADS product values for ecTRPV4^−/−^ and WT under basal conditions and following stimulation with ACh (lines indicate medians and pluses indicate means). * denotes *p* < 0.05. (**b**) Histograms of log ADS values show full composite event distributions under base (left) and ACh-stimulated (right) conditions; the bottom panel shows difference plots between the ecTRPV4^−/−^ and WT histograms.

Assessment of individual event parameters suggested a specific type of dynamic Ca^2+^ event may be deficient in ecTRPV4^−/−^ endothelium. In order to recapitulate holo-events, we combined normalized amplitude, duration and spatial spread parameters to generate a single index value for each event (the Amplitude Duration Spread product (ADS product)). Notably, this index exposes events that are essentially larger or smaller than the mean basal Ca^2+^ signal, which would have an ADS value of 1. Because calculated ADS values were highly left skewed (basal WT values raged from 0.05 to 171 with 75th percentile at 1.2 and ecTRPV4^−/−^ values ranged from 0.06 to 468 with 75th percentile at 12.1), they were plotted as log values for display and analysis (Figure 4); a log ADS value of 0 corresponds to an ADS value of 1. Overall, ADS values revealed a distinct difference in the core population of ecTRPV4^−/−^ and WT events (Figure 4a), with WT events being substantially smaller. Notably, stimulation with ACh caused a significant net increase in WT ADS values but had minimal impact on ecTRPV4^−/−^ ADS values, other than narrowing the range. Distribution of values as full histograms (Figure 4b) reveals the specific event population lacking in ecTRPV4^−/−^ endothelium (corresponding ADS values of 0.1 to 1.5). At the same time, the ecTRPV4^−/−^ endothelium exhibits a broad distribution of larger events lacking in WT endothelium (corresponding ADS values of 1.5 to 316). In WT endothelium, stimulation with ACh caused a distinct bimodal distribution of Ca^2+^ signals, whereby a population of small events (ADS ~0.14) persisted and a new population of larger events (ADS 2-8) emerged. On the contrary, stimulation of the ecTRPV4^−/−^ endothelium with ACh caused only expansion of the general basal distribution (ADS 2-8). Notably, although the differences between ecTRPV4^−/−^ and WT event populations narrow with ACh treatment, the same population of small events are lacking in the ecTRPV4^−/−^ endothelium.

## 3. Discussion

The TRP class of nonselective cation channels encompasses a diverse group of membrane proteins that play an essential role in cell signaling and homeostasis. Recent implication of Ca^2+^-permeable TRPV4 channels in cardiovascular disease has focused attention on their specific impact on vascular signaling and their potential as targets for therapeutic intervention. Studies pointing to TRPV4 channels as conduits of localized Ca^2+^ sparklets along plasma membranes of endothelial cells have suggested their role as key drivers of endothelial function [2,20,21], but their direct impact on the dynamic Ca^2+^ signaling profile in intact endothelium has remained obscure. Here, we provide evidence that endothelium-specific ablation of TRPV4 channels substantially reduces a particular population of small Ca^2+^ events along the vascular intima and right-shifts the Ca^2+^ signal profile to a single broad population that is monolithically preserved under endothelial stimulation. These findings imply that TRPV4 channels are crucial for maintaining the normal dynamic range of endothelial Ca^2+^ signals and a progressive pattern of signal recruitment during endothelial stimulation.

Selective ablation of endothelial TRPV4 channels allowed us to specifically address the impact of TRPV4 channels within the intima while preserving TRPV4 expression and function in other cell types, including vascular smooth muscle and perivascular nerves [5]. Indeed, we observed TRPV4 expression in the medial and adventitial layers of both ecTRPV4^−/−^ and WT arteries. This is particularly important since previous studies evaluating endothelial TRPV4 channels using agonists/antagonists or global TRPV4 knockout models may have included off-target effects [7,12,22]. We employed our custom autodetection/tracking algorithm LC_Pro in order to provide comprehensive and reproducible evaluation of a broad range of dynamic Ca^2+^ signals along the arterial intima. We found that endothelial TRPV4 ablation had little overall influence on the frequency of events occurring along the intima although the time course of ACh-induced event firing was distinctly abbreviated (occurring almost entirely within the first 60 s of exposure). These findings may suggest an important role for TRPV4 in sustained endothelial functional responses. A key finding of the current study was that removal of endothelial TRPV4 channels considerably altered the specific attributes (event parameters) of the dynamic endothelial Ca^2+^ signals. Most importantly, the number of events with amplitudes <1.2F/F_0_ and durations < 3.5 s were greatly reduced. Surprisingly, this loss of small-amplitude/duration events was accompanied by a relative increase in larger/longer-lasting events. The deficiency of small Ca^2+^ events in ecTRPV4^−/−^ endothelium is consistent with general loss of TRPV4 Ca^2+^ sparklets. In fact, the “missing” Ca^2+^ events in the ecTRPV4^−/−^ endothelium describe a discrete a population of small transients (amplitude ~0.12 ΔF above baseline, duration ~0.65 s, spatial spread ~16 µm^2^, and ADS ~0.3) quite similar to previously described TRPV4 Ca^2+^ sparklets (amplitude ~0.2 ΔF above baseline, duration (0.04–0.52 s), spatial spread (5–11 µm^2^) [2,20]. We might assume that the sparklet-type events measured here in the WT but not in ecTRPV4-ablated endothelium were those occurring in isolation that did not reach sufficient magnitude to induce Ca^2+^-induced Ca^2+^ release from internal stores. Indeed, the right-shifted mode we observed in the WT histogram (e.g., ADS) following ACh stimulation suggests conversion of some sparklet-type events to larger events, perhaps due to IP_3_-sensitzation of CICR. This is consistent with expansion of TRP-originating signals into Ca^2+^ waves as previously reported in intact arterial endothelium [2,23]. The source of the anomalous population of larger events in the ecTRPV4^−/−^ endothelium is unknown but may reflect a compensatory expansion of internal stores, perhaps through upregulation of IP3/IP3R signaling. Exploring this apparent adaptation is warranted given the known pathological implications of downregulated TRPV4 signaling. In the context of our current findings, it would appear that endothelial TRPV4 channels underlie a narrow range of physiological Ca^2+^ signals that may serve as important local regulators and key triggers for expanding Ca^2+^ signal profiles.

While amplitude, duration and spatial spread provide valuable and definitive information about individual Ca^2+^ events, each event is actually an amalgamation of all three parameters, and it cannot be assumed that any single event is actually represented by averaged individual parameters. Calculating the ADS product allowed us to gauge the relative “size” of each event (intensity in time and space) relative to the expected prototypical control event. Altogether, data suggest that in the absence of TRPV4 channels, the vascular endothelium largely loses the capacity to produce a specific event (ADS mode ~0.3) and may compensate by basally generating events that are an order of magnitude larger (primary ADS mode ~3). The later appears to be the same population of events recruited by normal endothelium upon stimulation (i.e., with ACh). This finding predicts that chronic loss of TRPV4 channels may cause an amplification of basal signaling that mimics an endothelium under constant (agonist-mediated) stimulation. Whether this translates to amplified Ca^2+^ impact on basal endothelial function in ecTRPV4^−/−^ (i.e., persistent vasodilation) or causes desensitization of Ca^2+^-dependent signaling (i.e., impaired vasodilator response) is an intriguing question for future study. The findings may also indicate that the characteristic shift from one predominant mode of event to another is a crucial aspect of graded stimulus-dependent signaling and that TRPV4 channels play a key role in this tuning of endothelial response.

## 4. Materials and Methods

### 4.1. Animals

Mice (equal number male and female, 25–35 g) were euthanized with pentobarbital sodium (50 mg/kg) and both common carotid arteries were harvested. All animal procedures were approved by the University of South Alabama Institutional Animal Care and Use Committee (1162322-3, 4 March, 2020; 1372680-4, 14 January 2020) and carried out in accordance with the NIH Guide for the Care and Use of Laboratory Animals.

### 4.2. Generation and Breeding of ecTRPV4^−/−^ Mice

Endothelium-specific TRPV4-deficient mice were produced by genetic targeting and cell-specific deletion as previously described [24]. Briefly, TRPV4 exon 13 was flanked with two loxP elements. A PacI digestion site was included upstream of the 5′ loxP site that allowed differentiation between the TRPV4-floxed allele (with PacI site) and the wild-type allele (without PacI site). Homozygous TRPV4-floxed (f/f) mice were generated. Mice were crossed with endothelium-specific CRE, and TRPV4f/f + CRE expressers were selected as breeders to create the TRPV4 knockout (^−/−^) genotype. Primer pairs used were sense 5′-CATGAAATCTGACCTCTTGTCCC and antisense 5′-TTGTGTACTGTCTGCACACCAGGC, which yielded ~2.4 kB PCR products.

### 4.3. Immunofluorescence Staining

Carotid artery segments were fixed in optimal cutting temperature compound (OCT) and then cut into 7 µm cross-sections. TRPV4 protein was labeled with rabbit TRPV4-specific primary antibody (Alomone Labs, Jerusalem, Israel) and fluorescent anti-rabbit secondary antibody (Alexa Fluor 647; red; Thermo Fisher Scientific, Waltham, MA USA). The elastic lamina autofluorescence was assessed using the green channel (488 nm excitation/510 nm emission). Digital images were collected in 6 µm stacks (0.5 µm increments) using a Nikon A1R confocal microscope and NIS Elements (Nikon Inc., Melville, NY, USA).

### 4.4. Ca^2+^ Imaging and Data Processing

Carotid artery segments were cut open longitudinally and mounted on sylgard inserts, intima side up, using tungsten micropins as previously described [25]. Arteries were incubated at room temperature for 40 min in the dark with Ca^2+^ indicator loading solution containing Fluo-4 AM (Thermo Fisher Scientific, Waltham, MA USA) in HEPES-buffered PSS (containing in mM 134 NaCl, 6 KCl,1 MgCls, 2 CaCl_2_, 10 HEPES, 10 glucose; pH 7.45). After washing and allowing 15 min for equilibration, inserts were placed in glass-bottom chambers (separated from coverglass by 100 μm) containing HEPES. The chamber was mounted on an inverted microscope of an Andor Revolution spinning disk confocal system (Andor, Belfast, UK). Ca^2+^-dependent fluorescence (488 nm excitation, 510 nm emission) was measured at 8 frames/sec at 25 ˚C (20× objective; 1024 × 1024 pixels) using iQ software (Oxford Instruments, Oxon, UK). The 16 bit raw data images were saved as 8 bit TIFF files and processed using the custom algorithm LC_Pro, implemented as a plug-in with ImageJ software (National Institutes of Health, Bethesda, Maryland, USA). A detailed description of the algorithm is provided in [26]. Briefly, this analysis software is specifically designed to: 1) detect sites of dynamic Ca^2+^ change above statistical (*p* < 0.01) noise, 2) track full event spatial boarders, 3) define circular regions of interest (ROI; 10 pixel or 3.4 µm diameter) at active site centers, and 4) analyze average fluorescence intensities within ROIs. Fluorescence data are expressed as F/F_0_, where F_0_ is determined by a linear regression of base data at each ROI. The amplitude is taken as the peak F/F_0_ for each event. The duration of each event is measured at 50% of the maximal amplitude. This approach prevents the arbitrary extension of duration values for events that decay to values very near the baseline. Events that do not reach 50% decay are assigned duration values to the end of the recording. Spatial spread of each event is determined from the maximal-dimension ellipse fit by the algorithm resulting from continuous event tracking.

### 4.5. Data Analysis

Normally distributed data are presented as the mean ± standard error. Non-Gaussian data are presented as distributed histograms or presented as box-and-whisker plots. For composite ADS values that represent the combined parameter attributes of each Ca^2+^ event, we normalized the amplitude (A), duration (D) and spatial spread (S) of each event to the control base mean value for each parameter and calculated the ADS product for each event. Statistical analysis was performed with GraphPad Prism software (GraphPad Software, San Diego, CA, USA) using a Mann–Whitney test (non-parametric) for single comparisons. *p* values < 0.05 were considered significant.

## 5. Conclusions

Genetic ablation of endothelial TRPV4 channels impairs the generation of small Ca^2+^ transients in the intact vascular endothelium and alters the normal pattern of Ca^2+^ signal recruitment by endothelial stimulation. These findings suggest an important role of endothelial TRPV4 channels in the regulation of Ca^2+^-dependent vascular homeostasis.

## Abbreviations

TRPV4	Transient receptor potential vanilloid 4 channel
ecTRPV4^−/−^	Endothelium-specific TRPV4 knockout mouse
ACh	Acetylcholine
IP_3_R	Inositol 1,4,5-trisphosphate
K_Ca_	Calcium-activated potassium channels
EL	Elastic lamina
WT	Wild type
CICR	Calcium-induced calcium release
GPCR	Gq protein-coupled receptor
ADS	Amplitude Duration Spread product
OCT	Optimal cutting temperature compound
